# Adaptive Signal Processing and Machine Learning Using Entropy and Information Theory

**DOI:** 10.3390/e24101430

**Published:** 2022-10-08

**Authors:** Tokunbo Ogunfunmi

**Affiliations:** Department of Electrical & Computer Engineering, Santa Clara University, Santa Clara, CA 95053, USA; togunfunmi@scu.edu

This Special Issue on “Adaptive Signal Processing and Machine Learning Using Entropy and Information Theory” was birthed from observations of the recent trend in the literature. Information theoretic learning (ITL) approaches have recently emerged as an effective solution to handle scenarios when we require specific models that fit the data, even in noisy and adverse conditions, and especially when the error distribution is non-Gaussian, such as in supervised learning.

The presence of strong disturbances in the error signal can severely deteriorate the convergence behavior of adaptive filters and, in some cases, cause learning algorithms to diverge. Entropy-based cost functions have replaced mean-square-error (MSE)-based ones and have been widely used in adaptive signal processing and machine learning to improve performance by designing and optimizing effective and specific models that fit the data, even in noisy and adverse conditions.

Our goal is to publish recent developments in the areas of adaptive signal processing, machine learning, and deep learning using information theory and entropy to improve performance in widespread and popular problems, and also to provide effective solutions to emerging problems.

These learning techniques rely on the paradigm of learning from data that have become indispensable tools for extracting information, making decisions, and interacting with our environment.

The scope of the Special Issue includes survey, theoretical and applications papers pertaining to all problems involving learning from data. We were able to garner articles submitted on a wide range of topics in this Special Issue. We published 11 papers in our Special Issue. Here, is a synopsis of the eleven papers:

In the first paper [1], titled “An Overview of Variational Autoencoders for Source Separation, Finance, and Bio-Signal Applications”, the authors present a general comprehensive overview of variational autoencoders and their applications. Autoencoders have found wide applications in dimensionality reduction, object detection, image classification, and image denoising applications. Variational autoencoders (VAEs) can be regarded as enhanced autoencoders where a Bayesian approach is used to learn the probability distribution of the input data. Variational inference methods are discussed before presenting the variational autoencoder. Later, problems and tradeoffs with the VAE are discussed. Several variants of VAEs and their limitations are discussed. As examples of applications of VAEs, the authors present three areas: financial, speech source separations and bio-signal applications. The application to finance is relatively new and rapidly growing, with great potential. Experimental results for speech source separations applications are also presented and discussed. The paper concludes with a summary and identifies possible areas of research in improving performance of VAEs in particular and deep generative models in general, of which VAEs and generative adversarial networks (GANs) are examples.

The second paper [2], titled “Multi-Class Classification of Medical Data Based on Neural Network Pruning and Information-Entropy Measures”, is about finding a suitable machine learning architecture for a specific task (e.g., for classification, clustering) for medical data based on commonly used data analysis techniques such as text mining, big data analytics, and data mining. In this work, the authors propose a machine learning model for the multi-class classification of medical data. This model comprises two components—a restricted Boltzmann machine and a classifier system. It uses a discriminant pruning method to select the most salient neurons in the hidden layer of the neural network, which implicitly leads to a selection of features for the input patterns that feed the classifier system.

The study aims to investigate whether information–entropy measures may provide evidence for guiding discriminative pruning in a neural network for medical data processing, particularly cancer research, by using three cancer databases: breast cancer, cervical cancer, and primary tumor. The authors aimed to investigate the post-training neuronal pruning methodology using dissimilarity measures inspired by the information–entropy theory; the results obtained after pruning the neural network were an improvement over the existing methods.

The third paper [3], titled “Generalizing the Balance Heuristic Estimator in Multiple Importance Sampling”, proposes a generic family of multiple importance sampling estimators.

The authors first revisit the celebrated balance heuristic estimator, a widely used Monte Carlo technique for the approximation of intractable integrals. Then, they establish a generalized framework for the combination of samples simulated from multiple proposals. Their approach is based on considering as free parameters both the sampling rates and the combination coefficients, which are the same in the balance heuristics estimator. Their framework contains the balance heuristic as a particular case. Therefore, their new family of estimators generalizes the balance heuristic. They address five cases of special interest, depending on which parameters are free and the number of samples simulated from each technique. Then, for these five examples, they find the variances for some notable selection of parameters showing that, for the important case of equal count of samples, their estimator with an optimal selection of parameters outperforms the classical balance heuristic. Finally, new heuristics are introduced that exploit the theoretical findings.

The authors in the fourth paper [4], titled “A Probabilistic Re-Interpretation of Confidence Scores in Multi-Exit Models”, propose an approach to train a deep neural network with multiple intermediate auxiliary classifiers branching from it. These so-called ‘multi-exits’ models can be used to reduce the inference time by performing an early exit on the intermediate branches if the confidence of the prediction is higher than a threshold. A critical assumption made here is that not all the samples require the same amount of processing to yield a good prediction.

The paper also proposes a way to jointly train all the branches of a multi-exit model without hyper-parameters by weighting the predictions from each branch with a trained confidence score. Each confidence score is an approximation to the real one produced by the branch, and it is calculated and regularized while training the rest of the model. They evaluate their proposal on a set of image classification benchmarks, using different neural models and early exit stopping criteria.

The authors in the fifth paper [5], titled “Ultra-Low-Power, High-Accuracy 434 MHz Indoor Positioning System for Smart Homes Leveraging Machine Learning Models”, propose and implement an intelligent, highly accurate and low-power indoor positioning system for smart homes, leveraging a Gaussian Process Regression (GPR) model using information–theoretic gain based on a reduction in differential entropy. The system is based on time difference of arrival (TDOA) and uses ultra-low-power radio transceivers working at 434 MHz. The system has been deployed and tested using indoor measurements for two-dimensional (2D) positioning. In addition, the proposed system provides dual functionality with the same wireless links used for receiving telemetry data, with configurable data rates of up to 600 Kbauds. The implemented system integrates the time difference pulses obtained from the differential circuitry to determine the radio frequency (RF) transmitter node positions. The implemented system provides a high positioning accuracy of 0.68 m and 1.08 m for outdoor and indoor localization, respectively, when using GPR machine learning models, and provides telemetry data reception of 250 Kbauds. The system enables low-power battery operation with consumption of <200 mW power with ultra-low-power CC1101 radio transceivers and additional circuits with a differential amplifier. The proposed system provides low-cost, low-power and high-accuracy indoor localization and is an essential element of public well-being in future smart homes.

The sixth paper [6], titled “An Adaptive Deblurring Vehicle Detection Method for High-Speed Moving Drones: Resistance to Shake”, proposes an improved GAN (generative adversarial networks) called Drone-GAN (which is an end-to-end adaptive vehicle detection algorithm (DCNet) for drones) to enhance the vehicle features of blurred images to be processed for unmanned aerial vehicles (UAVs).

A clarity evaluation module is used to adaptively determine whether the input image is a blurred image using improved information entropy. Extensive experiments were performed, the results of which show that the proposed method can detect both blurred and clear images well in poor environments (complex illumination and occlusion). The detector proposed achieves larger gains compared with state-of-the-art detectors. The proposed method can enhance the vehicle feature details in blurred images effectively and improve the detection accuracy of blurred aerial images, which shows good performance with regard to resistance to shake. 

The paper titled “A Novel Noise Reduction Method of UAV Magnetic Survey Data Based on CEEMDAN, Permutation Entropy, Correlation Coefficient and Wavelet Threshold Denoising” is the seventh paper [7], and it addresses the processing of unmanned aerial vehicle (UAV) magnetic data. The authors propose a noise-reduction method of UAV magnetic data based on complete ensemble empirical mode decomposition with adaptive noise (CEEMDAN), permutation entropy (PE), correlation coefficient and wavelet threshold denoising. First, the original signal is decomposed into several intrinsic mode functions (IMFs) by CEEMDAN, and the PE of each IMF is calculated. Second, IMFs are divided into four categories according to the quartiles of PE, namely noise IMFs, noise-dominant IMFs, signal-dominant IMFs, and signal IMFs. Then, the noise IMFs are removed, and correlation coefficients are used to identify the real signal-dominant IMFs. Finally, wavelet threshold denoising is applied to the real signal-dominant IMFs; the denoised signal can be obtained by combining the signal IMFs and the denoised IMFs. Both synthetic and field experiments are conducted to verify the effectiveness of the proposed method. The results show that the proposed method can eliminate the interference to a great extent, which lays a foundation for the further interpretation of UAV magnetic data.

The least-square twin K-class support vector machine (LST-KSVC) used in leak detection of water pipelines is a novel, simple and fast multi-classification method. However, LST-KSVC has a non-negligible drawback in that it assigns the same classification weights to leak samples, including outliers that affect classification; these outliers are often situated away from the main leak samples. To overcome this shortcoming, the maximum entropy (MaxEnt) version of the LST-KSVC, called the MLT-KSVC algorithm, is proposed in the eighth paper, titled “Leak Detection in Water Pipes Based on Maximum Entropy Version of Least Square Twin K-Class Support Vector Machine” [8]. In this classification approach, classification weights of leak samples are calculated based on the MaxEnt model. Different sample points are assigned different weights: large weights are assigned to primary leak samples and outliers are assigned small weights; hence, the outliers can be ignored in the classification process. Leak recognition experiments demonstrate that the proposed MLT-KSVC algorithm (compared with LST-KSVC, TwinSVC, TwinKSVC, and classic Multi-SVM algorithms) can reduce the impact of outliers on the classification process and avoid the misclassification color block drawback in linear LST-KSVC.

A major advantage of the use of passive sonar in the tracking of multiple underwater targets is that they can be kept covert, which reduces the risk of being attacked. However, the nonlinearity of the passive Doppler and bearing measurements, the range unobservability problem, and the complexity of data association between measurements and targets make the problem of underwater passive multiple target tracking challenging. To deal with these problems, the cardinalized probability hypothesis density (CPHD) recursion, which is based on Bayesian information theory, is developed to handle the data association uncertainty, and to acquire existing targets’ numbers and states (e.g., position and velocity) in the ninth paper [9] titled “Passive Tracking of Multiple Underwater Targets in Incomplete Detection and Clutter Environment”. The key idea of CPHD recursion is to simultaneously estimate the targets’ intensity and the probability distribution of the number of targets. CPHD recursion is the first moment approximation of the Bayesian multiple targets filter, which avoids the data association procedure between the targets and measurements including clutter. The Bayesian-filter-based extended Kalman filter (EKF) is applied to deal with the nonlinear bearing and Doppler measurements. The experimental results show that the EKF-based CPHD recursion works well in the underwater passive multiple target tracking system in cluttered and noisy environments

A novel variational autoencoder in the quaternion domain H, namely the QVAE, has recently been proposed, leveraging the augmented second order statics of H-proper signals.

The tenth paper [10], titled “An Information-Theoretic Perspective on Proper Quaternion Variational Autoencoders”, analyzes the QVAE under an information–theoretic perspective, studying the ability of the H-proper model to approximate improper distributions, as well as the built-in H-proper ones and the loss of entropy due to the improperness of the input signal. The authors conduct experiments on a substantial set of quaternion signals, for each of which the QVAE shows the ability of modelling the input distribution, while learning the improperness and increasing the entropy of the latent space. The proposed analysis demonstrates that proper QVAEs can be employed with a good approximation even when the quaternion input data are improper

In the fault monitoring of rotating machinery, the vibration signal of the bearing and gear in a complex operating environment has poor stationarity and high noise. How to accurately and efficiently identify various fault categories is a major challenge in rotary fault diagnosis.

Most of the existing methods only analyze the single channel vibration signal and do not comprehensively consider the multi-channel vibration signal.

In the final and eleventh paper [11], titled “A Refined Composite Multivariate Multiscale Fluctuation Dispersion Entropy and Its Application to Multivariate Signal of Rotating Machinery”, the authors present Refined Composite Multivariate Multiscale Fluctuation Dispersion Entropy (RCMMFDE), a method that extracts the recognition information of multi-channel signals with different scale factors, and the refined composite analysis ensures the recognition stability. Simulation results show that this method has the characteristics of low sensitivity to signal length and strong anti-noise ability. At the same time, combined with Joint Mutual Information Maximization (JMIM) and support vector machine (SVM), the RCMMFDE-JMIM-SVM fault diagnosis method has been proposed. This method uses RCMMFDE to extract the state characteristics of the multiple vibration signals of the rotary machine, and then uses the JMIM method to extract the sensitive characteristics. Finally, different states of the rotary machine are classified by SVM. The validity of the method is verified by the composite gear fault data set and bearing fault data set.

Overall, these eleven papers represent an outstanding perspective of current research in the areas covered in our original proposal. They show that the field is thriving and growing. We feature papers on recent developments in the areas of adaptive signal processing, machine learning, and deep learning using information theory and entropy to improve performance in widespread and popular problems, and also to provide effective solutions to emerging problems.

We hope you enjoy reading these papers.
